# Zinc transporter gene expression is regulated by pro-inflammatory cytokines: a potential role for zinc transporters in beta-cell apoptosis?

**DOI:** 10.1186/1472-6823-9-7

**Published:** 2009-02-25

**Authors:** Lærke Egefjord, Jens Ledet Jensen, Claus Heiner Bang-Berthelsen, Andreas Brønden Petersen, Kamille Smidt, Ole Schmitz, Allan Ertman Karlsen, Flemming Pociot, Fabrice Chimienti, Jørgen Rungby, Nils E Magnusson

**Affiliations:** 1 Department of Pharmacology, University of Aarhus, Aarhus, Denmark; 2 Department of Theoretical Statistics Department of Mathematical Sciences, University of Aarhus, Aarhus, Denmark; 3 Steno Diabetes Center, Gentofte, Denmark; 4 Diabetes Research Unit, Novo Nordisk A/S, Måløv, Denmark; 5 Mellitech, INAC/SCIB/, CEA Grenoble, France

## Abstract

**Background:**

β-cells are extremely rich in zinc and zinc homeostasis is regulated by zinc transporter proteins. β-cells are sensitive to cytokines, interleukin-1β (IL-1β) has been associated with β-cell dysfunction and -death in both type 1 and type 2 diabetes. This study explores the regulation of zinc transporters following cytokine exposure.

**Methods:**

The effects of cytokines IL-1β, interferon-γ (IFN-γ), and tumor necrosis factor-α (TNF-α) on zinc transporter gene expression were measured in INS-1-cells and rat pancreatic islets. Being the more sensitive transporter, we further explored ZnT8 (Slc30A8): the effect of ZnT8 over expression on cytokine induced apoptosis was investigated as well as expression of the insulin gene and two apoptosis associated genes, BAX and BCL2.

**Results:**

Our results showed a dynamic response of genes responsible for β-cell zinc homeostasis to cytokines: IL-1β down regulated a number of zinc-transporters, most strikingly ZnT8 in both islets and INS-1 cells. The effect was even more pronounced when mixing the cytokines. TNF-α had little effect on zinc transporter expression. IFN-γ down regulated a number of zinc transporters. Insulin expression was down regulated by all cytokines. ZnT8 over expressing cells were more sensitive to IL-1β induced apoptosis whereas no differences were observed with IFN-γ, TNF-α, or a mixture of cytokines.

**Conclusion:**

The zinc transporting system in β-cells is influenced by the exposure to cytokines. Particularly ZnT8, which has been associated with the development of diabetes, seems to be cytokine sensitive.

## Background

The zinc content of pancreatic β-cells is among the highest in the body [1] and zinc plays an important structural role in many proteins by binding protein molecules in dimers and, in the case of insulin, in oligomers. Insulin is stored as hexameric complexes chelating two zinc ions within vesicles in a crystalline state [2]. Furthermore, zinc is an important determinant of β-cell survival and a co-factor in metalloenzymes and zinc-dependent transcription factors [3,4]. Evidence that β-cell derived zinc is a major regulator of glucagon secretion is emerging [5,6]. Finally, the activity of β-cell L-type calcium channels, involved in insulin secretion, is partly regulated by zinc [7].

Diabetes affects zinc homeostasis [8] resulting in hypozincaemia, hyperzincuria and, most likely, a generalized zinc deficiency [2,9,10]. We have previously shown that the zinc content of β-cells is glucose dependent [11].

Regulation of zinc homeostasis is ensured by zinc transporters assigned to two metal transporter families: the ZIP proteins (SLC39a) and the ZnT proteins (SLC30a). ZIPs facilitate influx of zinc to the cytosol from the outside of cells or from the lumen of intracellular compartments while ZnTs ensure zinc efflux from cytosol to the outside of cells or to intracellular organelles [12]. The mammalian ZIP family comprises 14 proteins and the ZnT family 10 proteins, respectively [13]. The expression of a number of zinc transporters depend on ambient glucose concentrations [14,15]. Zinc levels are higher in ZnT8 over expressing cells with a higher zinc accumulation capacity and an enhanced glucose mediated insulin secretion. Furthermore, over expression of this protein has been reported to protect cultured β-cells from cell death induced by zinc depletion [16]. In the brain, the lack of specific zinc transporters, particularly ZnT3, is associated with apoptosis and amyloid deposition [17]. Sladek *et al. *[18] identified polymorphisms in the SLC30a8 gene encoding for the zinc transporter ZnT8 as a major genetic risk factor for the development of Type 2 diabetes, this was recently confirmed [19-21]. ZnT8 also appears to be a major humoral autoantigen involved in the pathogenesis of type 1 diabetes [22]. No genetic association between SLC30A8 and type 1 diabetes has been found [23,24].

Cytokines are well known mediators of cell death in the mature β-cell. This is due to the downstream signaling events orchestrated by the main inflammatory cytokines, TNF-α, IFN-γ, and IL1-β [25,26]. IL-1β alone or in combination with TNF-α and/or IFN-γ is toxic to β-cells in rat, mouse, and human islets and is in part mediated by transcriptional changes in the β-cells [27]. Destruction of β-cells is induced by highly reactive agents both oxygen derived free radicals and nitric oxide (NO) [8], which increase apoptosis [28] and decrease insulin production and release [29]. IL-1β is known to facilitate transcription of inducible NO-synthase (iNOS) by the activation of NFkB [30]. Data suggest that islets exposed to IL-1β have an impaired first phase insulin secretion [31]. Stimulation or over expression of different defense mechanisms protects β-cells against the toxic effect of cytokines [32-34]. Thus glucose responsive β-cells seem to be protected by anti apoptotic proteins. It has been shown that exposure to IL-1β and IFN-γ causes down regulation of the anti-apoptotic protein Bcl2 before the onset of apoptosis [35]. On the other hand, it has been established that the Bax protein promotes apoptosis in the β-cell [36].

Here we aim to explore whether cytokines influence zinc transporter expression and to provide a profile of the alterations. Having defined the more sensitive transporter, ZnT8, we explore the effects of over expression on the outcome of cytokine exposure to β-cells. We thus examine the hypothesis that the toxic effects of cytokines include regulation of zinc transporter genes.

## Methods

### Cell cultures

**Figure 1:**
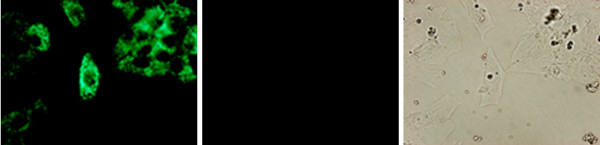
**Transfection of INS-1E cells with human ZnT8: Left panel shows fluorescence (green) marked ZnT8 RNA-probe positivity in transfected cells, contrasting the void control cells, middle panel**. Right panel shows the light microscopic appearance of the same cells.

INS-1 and INS-1E cells were cultured in a 5% CO_2 _atmosphere in complete RPMI 1640 supplemented with 11 mM glucose, 10% heat-inactivated fetal bovine serum, 50 μM beta-mercaptoethanol, 2 mM L-Glutamine, 100 U/mL penicillin, and 100 g/mL streptomycin. For stimulation assays cells were plated (10^6 ^cells/well) into 6-well plates (NUNC, Roskilde, Denmark). The INS-1 cells were treated with 60–180 U recombinant mouse IL-1β/mL, 200 U TNF-α/mL, 200 U recombinant Rat IFN-γ/mL or a mixture, consisting of 180 U IL-1β/mL, 200 U TNF-α/mL and 200 U Rat IFN-γ/mL (PharMingen International, San Diego, CA, USA). For INS-1 cells treatments were given for 1 and 24 hours, in replicas of 6. For INS-1E and INS-1E-ZnT8-EGFP cells treatments were given for 6 and 24 hours in replicas of 3. Controls were incubated with RPMI 1640 medium and 11 mM glucose. The stably transfected cell line INS-1E-ZnT8-EGFP was maintained as described above with the addition of 75 μM G418 to maintain a pure culture of cells expressing the ZnT8-EGFP construct. Expression of the fusion protein was controlled by fluorescence microscopy and by Q-PCR using ZnT8-EGFP specific primers (figure 1) [16].

### Rat islet preparation

Isolation and culturing of islets from 3–6 day old Wistar Furth rats (Charles River, Sulzfeldt, Germany) were as described [37]. The islets were precultured for 7 days in complete medium (RPMI 1640 with 100 U/mL penicillin and 100 μg/mL streptomycin and 11 mM glucose) supplemented with 10% newborn calf serum (Invitrogen/Gibco, Denmark). Five hundred randomly picked islets were used per 1 mL of complete medium supplemented with 0.5% human serum and 11 mM glucose. Islets were placed in 12-well plates (NUNC, Roskilde, Denmark), one plate for each time point. Cytokine treatments were given for 1, 2, 4, 6 and 24 hours in replicates of 4. Treatments were with 250 pg/mL IL-1β/mL, 10 ng/mL IFN-γ or a mixture, consisting of 250 pg/mL IL-1β and 10 ng/mL IFN-γ. Controls were incubated for 24 hours at identical conditions without addition of cytokines. At the end of the stimulation periods the islets were transferred to Eppendorf tubes for RNA isolation.

### Isolation of total RNA

For INS-1E cells total RNA was isolated using RNeasy Mini Kit 8 (Qiagen) according to the manufacturer's instructions. For archive material (islets and INS-1 cells) total RNA was isolated using TriZol. In brief, each sample was dissolved in 1 mL Trizol^® ^reagent (Invitrogen) on ice. Trizol was removed by addition of chloroform followed by isopropanol precipitation. The precipitates were washed using 75% ethanol. The amount and purity of total RNA was quantified using photospectrometry by measuring the optical density at 260 and 280 nm and the integrity was checked by agarose gel electrophoresis.

### Real time polymerase chain reaction

#### TaqMan assay

cDNA synthesis of archive (islets and INS-1 cells) material was performed using the RT-PCR TaqMan Kit N808-0234 (Perkin Elmer) according to the manufacturer's instructions. RefSeq IDs used for primer design and TaqMan IDs are listed in table 1. For the PCR-assay, 200 ng total RNA was used as starting material for the cDNA preparation in a reaction volume of 7.7 μL. Semi-quantitative RT-PCR was carried out in 20 μL reaction containing 2 μL TaqMan RT Buffer, 4.4 μL 25 mM MgCl2, 4 μL dNTP (200 μM), 1 μL random hexamer (100 pmol/μL), 0.4 μL RNAse Inhibitor, 0.5 μL MultiScribe Reverse Transcriptase (50 U/μL), 0.2 μL RNase free water and 7.7 μL RNA. Following reverse transcription cDNA solutions were incubated for 5 min at 95° to inactivate transcriptase and then stored at -20°.

**Table 1:** Primer sets used for real-time PCR; TaqMan assays were based on RefSeq ID.

Gene	RefSeq ID	TaqMan ID
ZnT3	NM_001013243.1	RN01472605_g1
ZnT4	NM_172066	RN01485635_m1
ZnT5	XM_226722.4	RN01493869_m1
ZnT6	XM_216643.4	RN01472402_m1
ZnT8	XM_001065623.1	RN01504406_m1
Zip5	XM_343140.3	RN01527166_g1
Zip6	NM_001024745	RN01405805_m1
beta-Actin	NM_031144.2	RN01412977_g1

RT-PCR was carried out in a volume of 20 μL per well in a 384-Well Optical Reaction Plates (A300990x) containing 1.0 μL TaqMan Expression Assay, 5.0 μL RNase free water, 10.0 μL TaqMan Universal PCR MasterMix (2×) (without AmpErase UNG) and 4.0 μL cDNA template. The plate was run on the Applied Biosystems 7900 HT Fast Real Time PCR System. The following protocol was used: 95° in 10 min for AmpliTaq Gold Enzyme Activation and finally the PCR including 40 cycles with 95° in 15 sec for denaturation and 60° in 1 min for annealing. To compensate for variation in cDNA concentrations and PCR efficiency between tubes, an endogenous control (beta-Actin) was included for each sample and used for normalisation.

#### SYBR Green assay

SYBR Green assay was used for experiments in INS-1E cells. Reverse transcription was performed on 500 ng total RNA for 1 h at 42°C by using a T7-oligo(dT)24 primer and Superscript II reverse transcriptase (Life Technologies). Cycling was performed using an ICycler from BioRad as previously described. Whenever possible the primers were placed in exons separated by an intron of at least 500 bp. To avoid pseudogenes, at least one of the primers was placed in a region distinct from these. To confirm that the primer pairs produced only unique products, a dissociation protocol was performed after thermo-cycling, thereby determining the dissociation of the PCR products from 65°C to 95°C. The real-time PCR assay included a no-template control and a standard curve of five serial dilution points (in steps of 10-fold) of a pool of the sampled cDNAs. All samples were amplified in duplicate.

#### Quantification

Normalised Ct 's were calculated by subtracting the Ct-mean of the normalisation genes for each sample and relative log2 fold changes were calculated using the comparative delta-delta C_t _method [38].

### Cell death assays

#### MTT assay

Cells were plated in 96-well plates and allowed to attach for 72 h. INS-1E and INS-1E-ZnT8-EGFP cells were then treated with cytokines or control condition (no addition of cytokines) for 6, 12, and 24 h. Each condition was performed in replicates of six. The experiment was repeated using cells grown from frozen stocks. The viability of each experimental condition was determined using the MTT [3-(4,5-dimethylthiazol-2-yl)-2,5-diphenyltetrazolium bromide] assay based on the activity of mitochondrial reductase enzymes. The assay was performed according to the manufacturer's instructions (Roche). Briefly, following experimental treatment, 10 μL of MTT solution was added to the culture medium and the plate was incubated in the dark for 3 hours at 37°C. A solubilisation buffer (100 μL) was added and allowed to incubate overnight at 37°C. Absorbance was measured at 570 nm using a ELX 808 microplate reader (Bio-Tek Instruments inc.). The percentage viability was calculated as follows: relative specific viability = [(A-B)/(C-B)] ×100 where A = ABS570 of the treated sample, B = ABS570 of the medium, and C = ABS570 of the control. The values were expressed as viability relative to control for each cell line.

#### Apoptosis/Necrosis assay

Cells were plated and treated as described in the MTT assay section and cell death was measured using the Cell Death Detection ELISA-plus assay (Roche) according to the manufacturer's instructions. Following experimental treatment culture supernatants were isolated and the cells were harvested in lysis buffer. After centrifugation culture supernatants and cell lysates were used to determine the amount of necrosis and apoptosis, respectively, in a quantitative sandwich-enzyme-immunoassay. Absorbance was measured at 405 nm and the viability was calculated as described in the MTT section. The assay was repeated twice as described above.

### Statistical analyses

Date are presented as means +/- s.e.m.. Anova analyses were performed to include a plate effect in INS-1 cells. Statistical significance of differences between stimulation and control was found using Student's t-tests. Anova analyses were performed on islet data. For the cell death assays results were considered significant at p < 0.01 in repeated experiments.

## Results

Significant differences between cytokine treated and control INS-1 cells are listed in table 2. Cytokines mostly down regulated zinc transporter expression. The down regulation in most cases increased over time. TNF-α treatment had almost no effect, whereas IL-1β, IFN-γ, and a mixture of cytokines all gave rise to down regulations of some of the genes. ZnT6 and ZnT8 were down regulated at both 1 and 24 hours, whereas ZnT3 was down regulated at 24 hours only. ZnT5 and Zip5 expression seemed not to be affected by cytokines and ZnT4 was only weakly influenced. Finally, Zip6 was influenced by IL-1β stimulation only, and at 24 hours only. A mixture of cytokines generally increased the down regulation.

**Table 2:** Regulation of ZnT/Zip expression in INS-1 cells following cytokine exposure.

	IL-1β/6 h	IL-1β/24 h	TNF-α/6 h	TNF-α/24 h	IFN-γ/6 h	IFN-γ/24 h	Mix./6 h	Mix./24 h	SEM
	
ZnT3	0.00	-0.58**	-0.14	0.00	-0.14	-0.93**	-0.14	-1.85**	+/- 0.20
ZnT4	-0.38	0.00	-0.14	0.49*	-0.49*	-0.38*	-0.49*	0.26	+/- 0.19
ZnT5	-0.14	0.00	0.00	0.26	-0.38	-0.38	-0.38	-0.26	+/- 0.23
ZnT6	-0.26**	-0.26**	-0.14	0.00	-0.26**	-0.26*	-0.14*	-0.14	+/- 0.09
ZnT8	-0.38*	0.26	-0.14	0.26	-0.49**	-0.38**	-0.49**	-1.81**	+/- 0.16
Zip5	0.00	-0.14	-0.14	0.38	0.14	-0.77*	-0.26	-0.38	+/- 0.36
Zip6	-0.00	-0.85**	0.00	0.00	-0.26	0.38*	-0.14	-1.00**	+/- 0.15

Log2-fold changes are indicated (n = 6). (* p < 0.05, ** p < 0.01). Mix. is IL-1β+TNF-α+IFN-γ IL-1β. The last column indicates standard errors +/- SEM for log2 fold changes.

**Figure 2:**
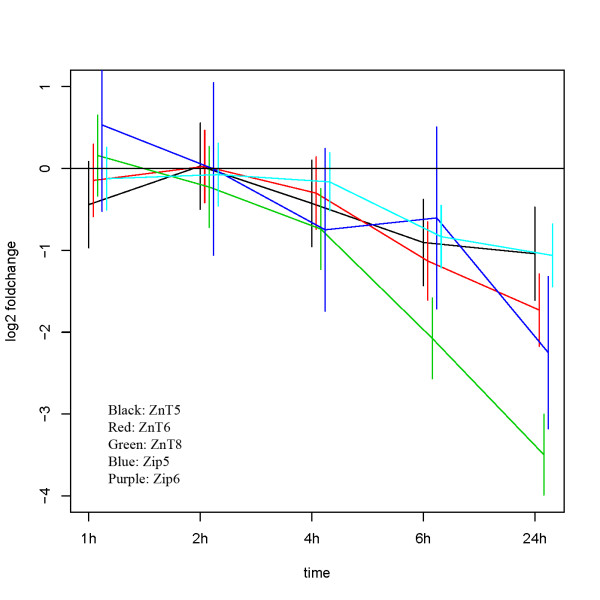
**Zinc transporter expression profiles for ZnT5, ZnT6, ZnT8, Zip5, and Zip6 following cytokine exposure in islets**. The figure shows the time course for the log2 difference +/- SEM between the IL-1β stimulation and the IFN-γ stimulation, showing a systematic development over time for the indicated genes. (n = 4). Black; ZnT5, red; ZnT6, green; ZnT8, blue; Zip5, purple; Zip6.

In islets there was no change over time for IFN-γ stimulation, whereas ZnT6 and ZnT8 showed significant changes over time for both IL-1β and the stimulation with IL-1β+ IFN-γ (table 3). There were significant differences between the three treatments at 24 hours for ZnT5, ZnT6 and ZnT8 (table 4). Figure 2 shows the time course for the difference between the IL-1β stimulation and the IFN-γ stimulation for ZnT5, ZnT6, ZnT8, Zip5 and Zip6. For these genes there was a systematic development over time. As in INS-1 cells a mixture of cytokines generally increased the down regulation. Cytokine stimulation for 24 hours resulted in an overall down regulation of the insulin gene in islets and INS-1 cells (results not shown). As for INS-1 cells, ZnT8 was the most regulated zinc transporter in islets where it was down regulated approximately 12 fold (~3.5 on a log2 scale).

**Table 3:** Regulation of ZnT/Zip expression in islets following cytokine exposure (n = 4).

	IL-1β	IFN-γ	IL-1β+ IFN-γ
ZnT3	0.7*	30.5	38.9
ZnT4	86.3	62.9	17.9
ZnT5	7.0	67.7	0.1*
ZnT6	1.40*	31.7	1.4*
ZnT8	0.0*	50.9	0.0*
Zip5	14.6	88.1	52.8
Zip6	27.0	55.4	41.5

P-values in percent for the hypothesis of no change over time are given. (*) p-values below 5% are highlighted.

**Table 4:** Regulation of ZnT/Zip expression in islets following cytokine exposure (n = 4).

	1 h	2 h	4 h	6 h	24 h
ZnT3	87.7	51.3	11.5	1.6*	25.2
ZnT4	40.8	69.8	54.0	36.9	25.8
ZnT5	62.5	99.3	50.9	22.4	1.1*
ZnT6	86.9	80.6	77.3	18.0	1.1*
ZnT8	76.5	75.7	46.3	0.8*	0.0*
Zip5	82.7	84.7	74.2	62.4	10.9
Zip6	55.6	90.8	80.3	26.5	5.0

P-values in percent for the hypothesis of no difference between treatments are given. (*) p-values below 5% are highlighted.

**Figure 3:**
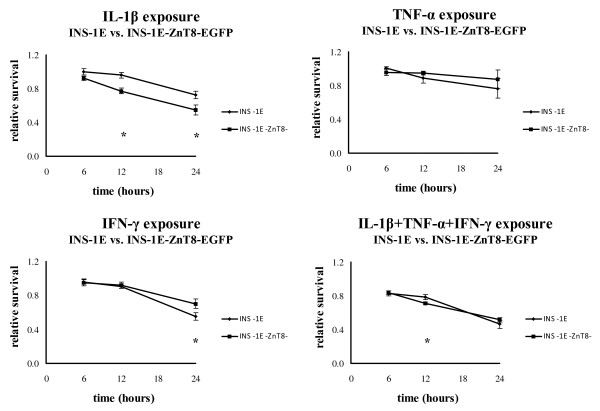
**Relative survival for INS-1E and INS-1E-ZnT8-EGFP cells following cytokine exposure after 6, 12, and 24 hours was estimatedby MTT**. Values were calculated relative to control cells for each cell line +/- SEM (n = 6). (*) p < 0.01 indicates significance in two independent experiments.

**Figure 4:**
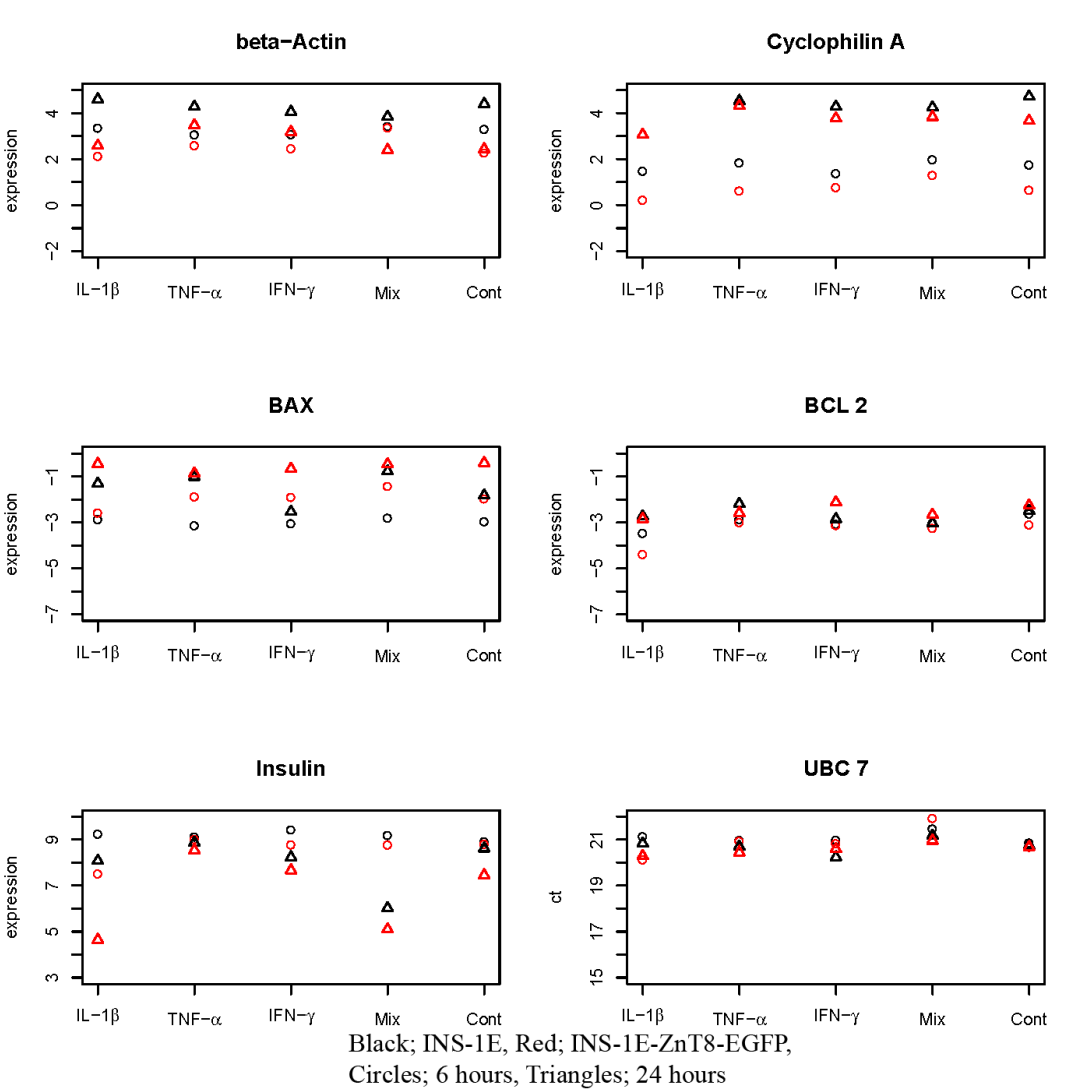
**Log2 expression levels relative to UBC7 for beta-Actin, Cyclophilin A, BAX, BCL2, and insulin in INS-1E and INS-1E-ZnT8-EGFP cells (n = 3)**. The last plot indicates the expression level of the normalizing gene UBC7. Black; INS-1E cells, red; INS-1E-ZnT8-EGFP cells. Circles; 6 hour treatments, triangles; 24 hour treatments.

The sensitivity to cytokines in INS-1E and INS-1E-ZnT8-EGFP cells was estimated by measuring mitochondrial activity (figure 3). No differences between the cell lines were found after exposure to TNF-α. Compared to INS-1E, IFN-γ decreased survival after 6 hours (but not after 12 or 24 hours). IL-1β decreased survival after 12 and 24 hours. Cytokine mixtures decreased survival after 6 hours (but not after 12 or 24 hours). These results were verified by an independent assay specifically measuring apoptosis and necrosis at 12, 24, and 36 hours with IL-1β and cytokine mixture. Apoptosis was increased by 4 and 2 fold in the ZnT8 transfected cell line following treatment with IL-1β for 24 and 36 hours, respectively (table 5). No differences were observed between the cell lines using the cytokine mixture. In addition, BCL2 and BAX mRNA expressions were compared between the cell lines at 6 and 24 hours (figure 4). Two of the housekeeping genes (beta-Actin and Cyclophilin A) showed a marked difference between the two cell lines and the two time points. Expression of the housekeeping gene UBC7 showed no difference between the two cell lines and a minor difference between the two time points. None of the housekeeping genes showed a difference between the treatments. UBC7 was therefore used as the normalizing gene. BAX and BCL2 showed no response to cytokine treatment (p = 0.07 and p = 0.13), but both genes showed a strong time effect (p < 0.0001 and p = 0.0001), and BAX exhibited a higher expression in the INS-1E-ZnT8-EGFP cells (p < 0.0001). Insulin gene expression showed a difference between the two cell lines and a difference between the two time points. On top of that IL-1β decreased the expression of insulin in INS-1E-ZnT8-EGFP cells (p = 0.0001) and a mixture of cytokines decreased the expression for both cell lines (p < 0.0001).

**Table 5:** Induction of apoptosis/necrosis in INS-1E-ZnT8-EGFP cells relative to INS-1E cells following cytokine exposure (n = 3).

	IL-1β/12 h	IL-1β/24 h	IL-1β/36 h	Mix/12 h	Mix/24 h	Mix/36 h
Apoptosis	-	*4.1 +/- 0.21	*1.8 +/- 0.23	-	-	-
Necrosis	-	-	-	-	-	-

Data represent mean of two independent experiments. Fold changes between cell lines are indicated. (*) p < 0.05 in both experiments. Only significant changes are shown +/- SEM. Mix. is IL-1β+TNF-α+IFN-γ IL-1β.

## Discussion

The present study demonstrates that cytokines regulate the expression of zinc transporter mRNA in β-cells. This may relate to both loss of cell mass (as is the case in the central nervous system [17]) and to decreased secretory capacity [16]. Evidence that zinc homeostasis is important for the development of diabetes is emerging from a number of association studies [18-20,22]. Zinc is concentrated in islet cells and related to insulin synthesis, storage and secretion [39]. Hypozincemia is a common feature in diabetes [40,41] and zinc supplementation has been shown to inhibit the development of experimental type 1 diabetes in mice [42]. Moreover, zinc can improve hyperglycemia in streptozotocin-diabetic mice [34]. We have previously described that some, but not all, ZnTs and ZIPs are influenced by ambient glucose levels and zinc concentrations in β-cells, suggesting an active role for these proteins. ZnT3 and ZnT8 appear to be of particular interest [14,15].

In the present study, cytokines generally down regulated the expression of zinc transporters in both islets and INS-1 cells and we found an increased regulation over time. In most cases, zinc transporter expression was similar for INS-1 cells and islets but of greater magnitude in islets. Some of the discrepancies may be due to the presence of other cell types in islets. Also, TNF-α was not tested in islets limiting the conclusions for this cytokine. Comparing individual cytokines showed that some zinc transporters, namely ZnT5, ZnT6 and ZnT8, exhibited similar expression profiles indicating that these transporters may share regulatory transcriptional mechanisms [43]. The mixture of cytokines, mimicking the *in vivo *cytokine load in islet inflammation, augmented the responses seen with the individual cytokines. Notably ZnT8 responded with very large significant down regulations to both IL-1β and the cytokine mixture. Excessive apoptosis of pancreatic β-cells has been associated with diabetes [44]. It has been shown that zinc depletion by itself can induce apoptosis [45] and it may also promote apoptosis induced by oxidative stress [46], thereby participating in reduction of β-cell mass. Enhanced capacity of the β-cell to store zinc may therefore protect against zinc depletion and oxidative stress [16]. On the other hand, some of the changes in intracellular zinc concentrations that can be expected from the changes in transporter expression described here might increase the intracellular load of free zinc ions and may add yet another mechanism for cell death since it has previously been demonstrated that an exaggerated trans-membrane zinc transport can severely affect beta-cell survival by a direct toxic effect of zinc [47].

Evidence suggests that ZnT8 enhances zinc storage in insulin granules and is directly implicated in the insulin secretion pathway. In this study we found that INS-1E cells over expressing ZnT8 were more sensitive to IL-1β induced apoptosis. Interestingly, compared to wild type cells, the cytokines IFN-γ and TNF-α as well as the cytokine mixture did not result in increased apoptosis, suggesting that ZnT8 may be affected by signalling pathways regulated by IL-1β. Palmer et al. [48] showed that sensitivity towards IL-1β depends on the metabolic status of the β-cell indicating that over expression of ZnT8 may increase the metabolic activity. Hence, contrasting previous studies, over expression of ZnT8 was not protective with respect to cytokine exposure. However, siRNA knockdown of this gene seems to increase the sensitivity of INS-1E cells (unpublished observation) suggesting that the level of protein and specific stressor are important for the outcome. Cytokines mediate β-cell death by varying pathways and are instrumental in the reduction of β-cell mass in both type 1 and type 2 diabetes [49]. β-cell destruction is the result of islet infiltration and an inflammatory response elicited by secretion of pro-inflammatory cytokines and chemotactic factors leading to insulinopenia and hyperglycemia [50]. Both IL-1β and TNF-α contribute to pancreatic β-cell death in type 1 diabetes probably via activation of transcription factor nuclear factor-kappa-B (NF-kappaB). IL-1β is associated with a more severe induction of cell death compared to TNF-α and was also observed in the present study (data not shown). Recent studies suggest that these differences may partly be explained by a stronger induction of NF-kappaB and its target genes by IL-1β [51]. The ZnT8 over expressing cell line exhibited a higher basal level of apoptosis compared to control cells indicating an increased pro-apoptotic environment *per se*. In line with this, the relative expression of BAX (pro-apoptotic) was higher in the transfected cell line and no difference was found for BCL2 (anti-apoptotic). Expressions of the insulin gene were significantly down regulated by similar magnitudes in both cell lines corroborating previous observations [29].

## Conclusion

In summary, the present study demonstrates for the first time that regulation of zinc transporters is sensitive to pro-inflammatory cytokines.

These data suggest that cytokines might contribute to the disturbed intracellular zinc homeostasis seen in β-cells of type 1 and type 2 diabetic patients. Furthermore, over expression of ZnT8 may sensitize INS-1E cells to apoptosis via IL-1β indicating a possible role for this protein in β-cell apoptosis. However, further mechanistic studies should be performed to determine the specific role for ZnT8 in the downstream events from IL-1β causing β-cell apoptosis.

## Competing interests

The authors declare that they have no competing interests.

## Authors' contributions

NEM and LE contributed to the conception, design, acquisition and analysis of data and wrote the manuscript. JLJ did the mathematical/statistical analyses. NEM and JLJ interpreted the results. CHB contributed to acquisition of data and made suggestions to the manuscript. ABP and KS contributed to acquisition of data. AEK, FP, FC, OS and JR contributed to the conception and made improvements to the manuscript.

## Pre-publication history

The pre-publication history for this paper can be accessed here:


